# Early Alterations of Cytoskeletal Proteins Induced by Radiation Therapy in the Parenchymal Cells of Rat Major Salivary Glands: A Comparative Immunohistochemical Analysis

**DOI:** 10.7759/cureus.75634

**Published:** 2024-12-13

**Authors:** Sherif S Hassan, Mashael S Alqahtani

**Affiliations:** 1 Department of Basic and Clinical Oral Sciences, Umm Al-Qura University, Makkah, SAU

**Keywords:** cytokeratin 17, parotid gland, radiotherapy, sublingual, submandibular

## Abstract

Objectives: Head and neck malignancies (HNMs) encompass a variety of cancers that affect the oral and para-oral tissues, the most common of which are squamous cell carcinomas. Radiotherapy is commonly used to treat these cancers, often involving radiation exposure to the salivary glands. This study aims to investigate the early impacts of radiotherapy on the internal microstructure of the salivary gland cells and identify which gland exhibits the highest level of radiosensitivity.

Methods: Twelve male albino rats were divided into two groups (control group and experimental group subjected to radiotherapy). The experimental group underwent a daily dose of 5 Grays of radiotherapy for six days with a total dose of 30 Grays, targeting the salivary glands. One month later, the salivary gland complex was dissected and processed for histological analysis and cytokeratin 17 (CK17) immunostaining.

Results: Histological examination of the irradiated salivary glands revealed atrophic changes in the gland parenchyma, accompanied by the proliferation of dense fibrous stroma. The parenchymal components consisted of small serous acini with poorly defined lumens, many of which had been replaced by fatty tissue with the formation of duct-like structures. Immunohistochemical findings of control glands exhibited weak to mild CK17 expression in duct cells, with the staining pattern typically diffuse or localized to the basal region of the cell. Some serous acini and serous demilunes within mixed acini exhibited diffuse, weak to mild CK17 expression, whereas the mucous acinar cells showed no expression. Radiated major glands revealed moderate to strong CK17 expression in the duct and serous acinar cells (p<0.05). Two distinct expression patterns were identified: the first exhibited diffuse expression across the cells, while the second showed intense expression at the apical region with mild expression at the basal part.

Conclusions: The positive immunostaining in control salivary glands suggests that small amounts of intermediate filaments are essential for saliva secretion. Radiotherapy significantly disrupts the cytokeratin arrangement and density in major salivary glands, impairing saliva production. The most prominent changes occurred in the submandibular gland, followed by the parotid gland, with the sublingual gland showing the least impact. However, CK17 intensity in acinar cells was highest in the parotid, then submandibular, and lowest in the sublingual gland. Further research is needed to assess the short- and long-term effects and potential for recovery over time.

## Introduction

Head and neck malignancies (HNMs) encompass a varied group of tumors that make up around 5% of all cancers in the body [1]. They are the seventh most common type of cancer, with more than 660,000 new cases diagnosed annually with a mortality rate of nearly 375,000 per year [2-3]. About 90% of HNMs are squamous cell carcinomas affecting the oral cavity, pharynx, larynx, and paranasal sinuses [4]. The salivary glands of rats include three pairs of major glands: the parotid gland, submandibular gland, and sublingual gland, along with numerous small salivary glands distributed throughout the oral cavity. Saliva is primarily composed of secretions from the submandibular glands (65%), parotid glands (23%), and sublingual glands (4%), with the remaining coming from various minor glands [5]. Saliva plays a crucial role in oral health by lubricating the oral mucous membranes, making it easier to chew and swallow food [6]. Saliva also contains enzymes that begin the digestive process and antimicrobial properties that protect against infections [7]. Additionally, the constant flow of saliva helps to neutralize acids produced by bacteria, reducing the risk of tooth decay [8].

Radiotherapy is commonly employed to treat HNMs, often including the salivary glands within the target zone [9]. Radiotherapy enhances clinical, aesthetic, and functional outcomes for cancer patients, whether as a primary treatment or as an adjunct to surgery eliminating any cancer remnants [9-10]. However, radiotherapy leads to unavoidable side effects on salivary gland tissue. The early side effects occur during or shortly after treatment and impact saliva production, taste perception, and oral mucosa, and the long-term side effects may emerge months or years later, affecting teeth, bones, and muscles, and delaying tooth eruption [11-15].

The cell's cytoplasm houses a three-dimensional filamentous network known as the cytoskeleton, which consists of three primary components: microtubules with a diameter of 25 µm, intermediate filaments measuring 6 to 10 µm, and microfilaments ranging from 4 to 6 µm in diameter [16]. Animal cells have been identified with six major intermediate filaments: cytokeratin, vimentin, desmin, glial fibrillary acidic protein, neurofilaments, and nuclear lamins [17]. Cytokeratin intermediate filaments are a group of related proteins present in the epithelial cells. They function in maintaining cellular structure, connecting cells, aiding the movement of cytoplasmic organelles, facilitating the transport of substances within the cell, and playing a crucial role in preserving the tensile strength and integrity of epithelial tissue [18].

Immunohistochemistry is a staining technique that identifies intracellular microstructures through the antigen-antibody reaction [19]. This approach allows for the localization of target molecules within their specific environments. Once the antibodies attach to the antigens in the tissue sample, the enzyme or dye becomes activated, making the antigens visible under the microscope [20]. IgG, commonly utilized in immunohistochemistry, is generated by immunizing an animal with a specific antigen, prompting a humoral immune response that produces a monoclonal antibody (anti-epitope) for isolating and detecting the expression of that antigen in cells [21]. Cytokeratin intermediate filaments are a group of proteins encoded by distinct genes and are expressed in various types of epithelial cells, including salivary gland parenchyma. Cytokeratin immunoreactivity is an important biomarker due to its strong antigenicity, stability, and consistent expression patterns [21].

Our study investigates the immunohistochemical expression of cytokeratin 17 (CK17) in the parenchyma of major salivary glands after fractionated radiotherapy to explore the differences in its effects across these glands.

## Materials and methods

**Figure 1:**
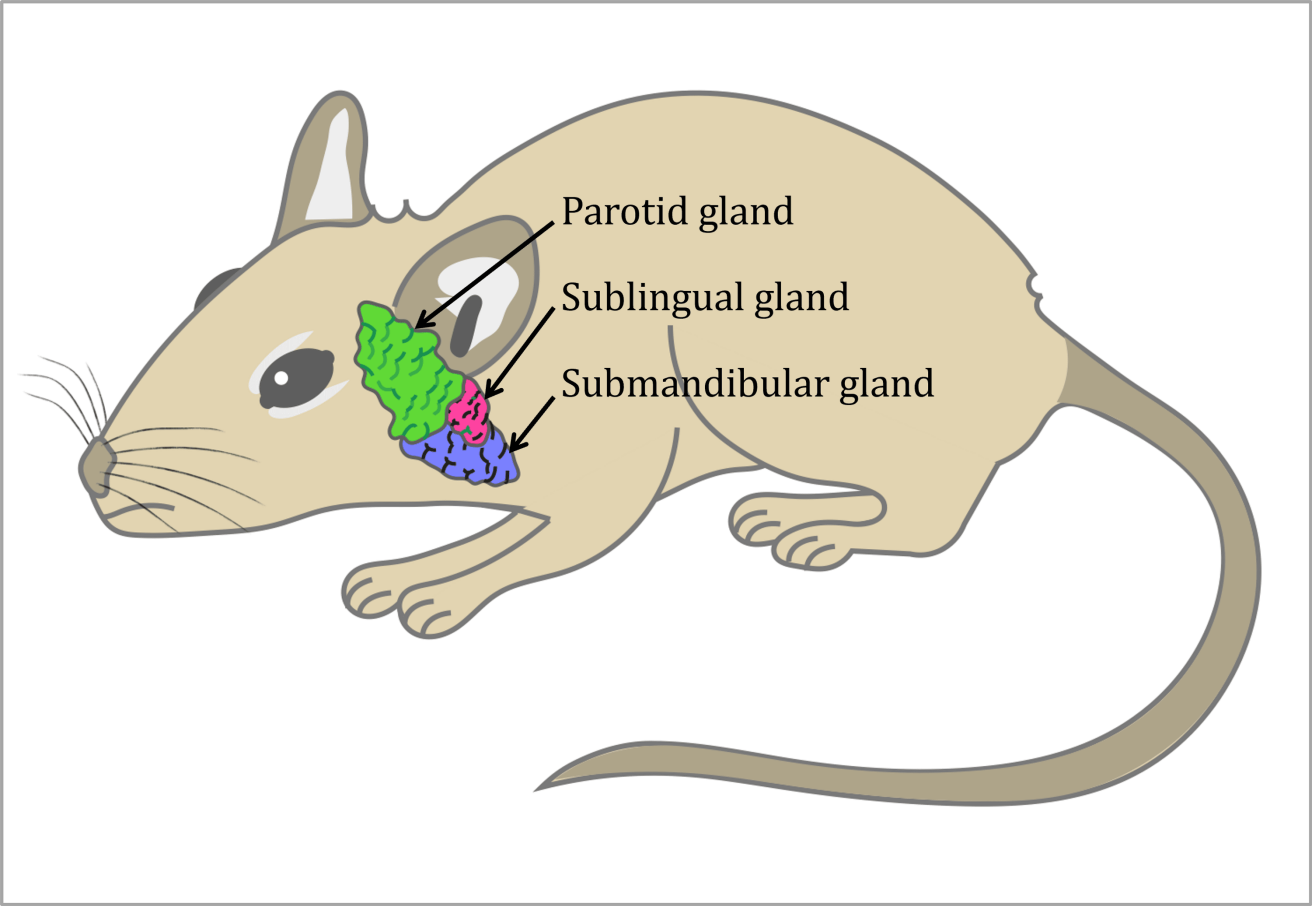
Location of the salivary gland complex of the rats Credit: Image created by the author

Twelve male albino rats weighing nearly 180 grams were included in the study and housed in an animal health care facility overseen by the Laboratory Animal House at the Faculty of Veterinary Medicine, Cairo University, Egypt (Figure 1). They were supplied with a mixture of hard and soft foods and open access to water. The rats were divided into a control group and an experimental group. The experimental group received daily radiotherapy of 5 Grays for six consecutive days with a total dose of 30 Grays [15,18].

Ethical consideration

Ethical approval was obtained from the Biomedical Research Ethics Committee of the Faculty of Dentistry, Al-Azhar University, Egypt, under approval no. AUARE 0001-2/2023.

Radiation exposure

On the days of radiation, rats of all groups were administered general anesthesia using 30 mg/kg of thiopental sodium (Egyptian Pharmaceutical Company, Egypt). The experimental rats were protected with a 5 mm thick lead sheet to shield the surrounding vital organs. The exposed neck area received a radiation dose of 5 Grays (1 Gy/min) from 8:00 am to 12:00 pm for six consecutive days, utilizing a therapeutic X-ray beam (Philips SL 75.5, Philips Medical Systems, United Kingdom) operating at 235 kV and 15 mA, with a maximum field size of 40 x 40 mm at 43 cm from the focus. One month after the final dose, the rats were euthanized with a pentobarbital injection. The salivary gland complex was carefully dissected and fixed in 10% neutral buffered formalin for three days. The paraffin-embedded tissue sections were then processed for staining with hematoxylin and eosin for traditional histopathological examinations.

Immunostaining technique

Paraffin sections of 5-6 μm were placed on silicone-coated glass slides, deparaffinized with xylene, and rehydrated through decreasing ethanol concentrations. Endogenous peroxidase activity was then blocked using methanol containing 0.3% hydrogen peroxide. Subsequently, antigen retrieval was carried out in a microwave for five minutes, after which the slides were immersed in a blocking reagent for 10 minutes to reduce nonspecific staining. CK17 was detected using the monoclonal anti-CK17 E3 antibody (Sigma, Germany) with a labeled streptavidin-biotin (LSAB) method and hematoxylin (counterstain). The positive staining reaction appeared as a brownish hue, reflecting the intracellular distribution of CK17 within the duct and acinar cells. Staining intensity was assessed quantitatively, with scores ranging from negative (0) to strong (4).

The statistical analysis aims to determine if there are significant differences in CK17 intensity between the control and the radiated glands and to identify which glands are most affected by radiation. Data analysis was performed using IBM SPSS Statistics for Windows, Version 23 (Released 2015; IBM Corp., Armonk, New York, United States). Quantitative data were presented as mean ± standard deviation (SD) to assess normal distribution. Levene’s test for equality of variances was performed as an initial step to evaluate the homogeneity of variances between the two groups. Independent t-tests were used to compare quantitative data across the groups. A 95% confidence interval was used, with a 5% margin of error, and a p-value of ≤0.05 was considered statistically significant. The gland with the lowest p-value indicated the most notable difference in cytokeratin intensity between the control and radiated groups.

## Results

All animals in the experiment remained healthy until the time of sacrifice. Any early deaths that occurred on the day of radiation were attributed to anesthesia-related complications and excluded from the study.

The histopathological findings of the parotid gland of the control group displayed parenchymal lobular tissue filled with densely packed serous acini and a duct system, supported by connective tissue stroma that separates it into lobes and lobules. The control submandibular gland was characterized by predominantly serous acini with a few mucous acini, many of which were covered by serous demilune with a normal branching duct system. The control sublingual gland primarily contained mucous acini, many also covered by serous demilune, and a small number of serous spherical acini with normal branching ducts. Salivary gland elements in the animal subjected to radiotherapy exhibited atrophic changes, marked by fewer parenchymal components and loss of gland architecture with dense fibrous stroma. The parenchymal elements included small serous acini with undefined lumens, many of which were replaced by adipose tissue. Submandibular and sublingual glands of the second group exhibited a prominent presence of mucous acini. The duct systems displayed enlarged and dilated lumens, along with the presence of few duct-like structures. Histological analysis of the irradiated group indicated that the sublingual gland exhibited greater resistance to radiation.

**Figure 2:**
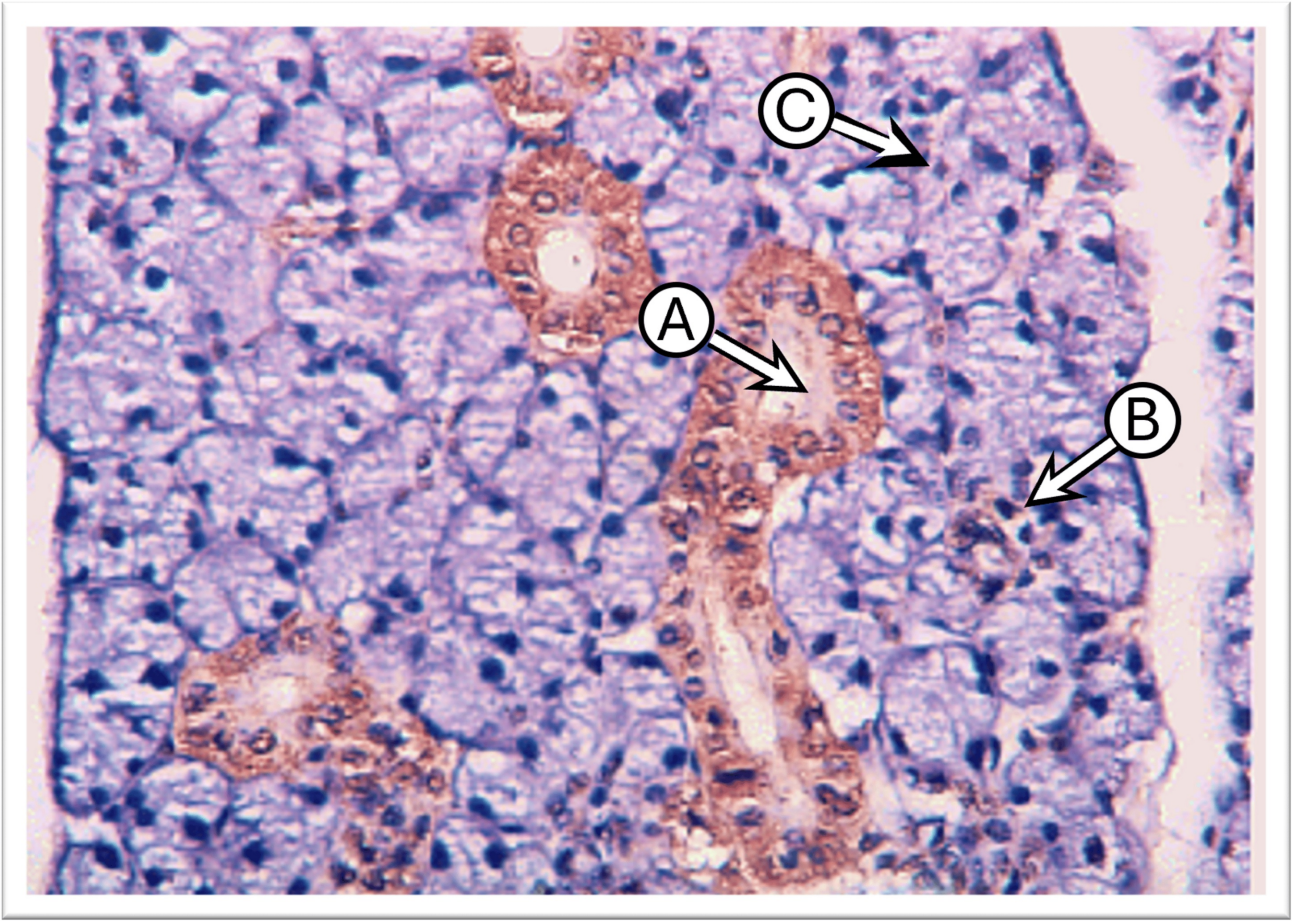
Photomicrograph of the parotid gland of the control group shows moderate CK17 staining at the basal part and weak at the apical part in the duct cells “A,” weak in some serous acini “B,” and negative in most serous acini “C” (immunoperoxidase technique x200 magnification)

The examination of control parotid gland sections incubated with anti-cytokeratin E3 antibody against CK17 showed a weak to mild expression in the intercalated, striated ducts and serous acinar cells. The cytokeratin intensity was lateral and basal to the nucleus with weak expression at the apical part of the cell cytoplasm. Many sections demonstrated diffuse staining patterns of CK17 in both duct and serous acinar cells. The main excretory ducts lined by stratified squamous epithelium displayed moderate expression at their basal layer with weak expression at the luminal layers (Figure 2).

**Figure 3:**
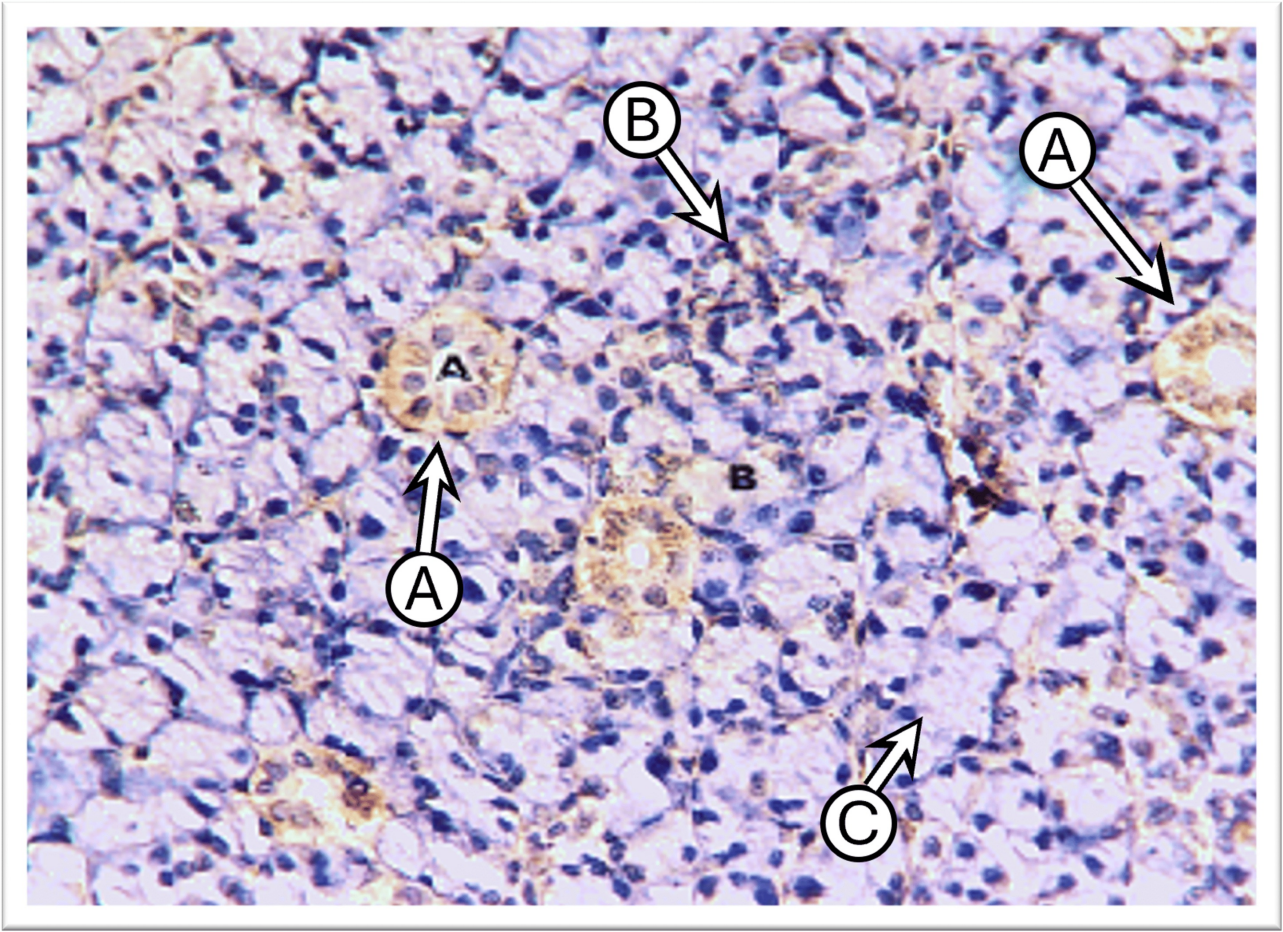
Photomicrograph of the submandibular gland of the control group shows mild diffuse CK17 staining in the duct cells “A,” weak in granular convoluting tubules “B,” and negative in serous acini “C” (immunoperoxidase technique x100 magnification)

The examination of control submandibular gland sections incubated with anti-cytokeratin E3 antibody targeting CK17 revealed weak to mild expression across various cell types. This included cells in the intercalated, striated, and excretory ducts and serous acinar cells, whereas mucous acini showed no staining. The staining pattern was either generally diffuse and uniform or concentrated laterally and basally around the nucleus, with weaker expression observed at the apical regions of the cytoplasm. In some sections, the main excretory duct lined by stratified squamous epithelium exhibited moderate expression at the basal layer with weak expression in the more superficial layers. The cells of the granular convoluted tubules displayed weak to mild staining in a diffuse pattern (Figure 3).

**Figure 4:**
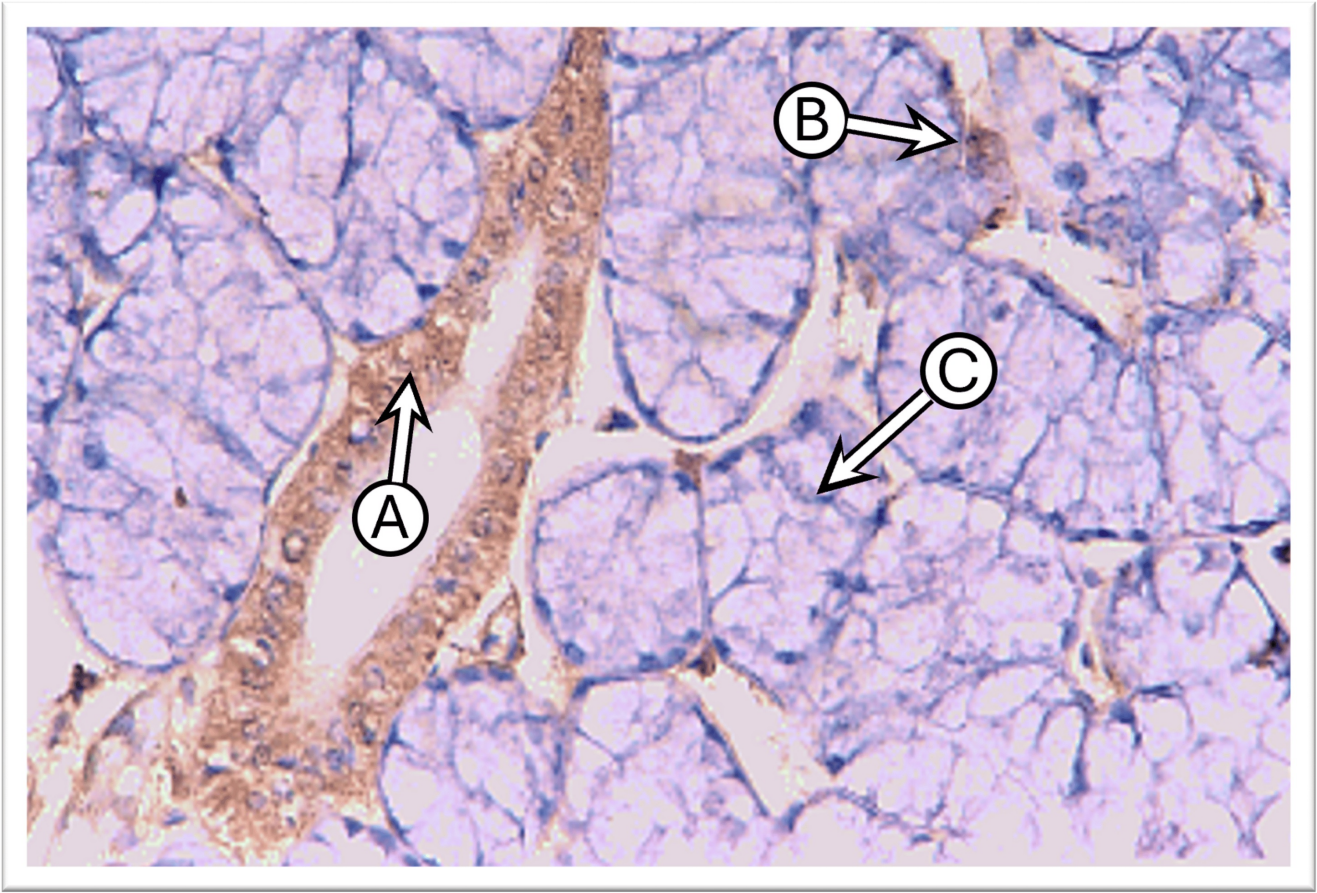
Photomicrograph of the sublingual gland of the control group shows moderate diffuse CK17 staining in the striated duct “A,” weak in serous acini “B,” and negative in mucous acini “C” (immunoperoxidase technique x400 magnification)

Control sublingual gland sections, incubated with the anti-cytokeratin E3 antibody targeting CK17, showed weak to mild expression in duct cells and in the serous demilune cells that cap the mucous acini, while mucous acini exhibited no staining. The staining pattern was predominantly diffuse and uniform or tended to concentrate laterally and basally around the nucleus, with weaker expression observed at the apical regions of the cells (Figure 4).

**Figure 5:**
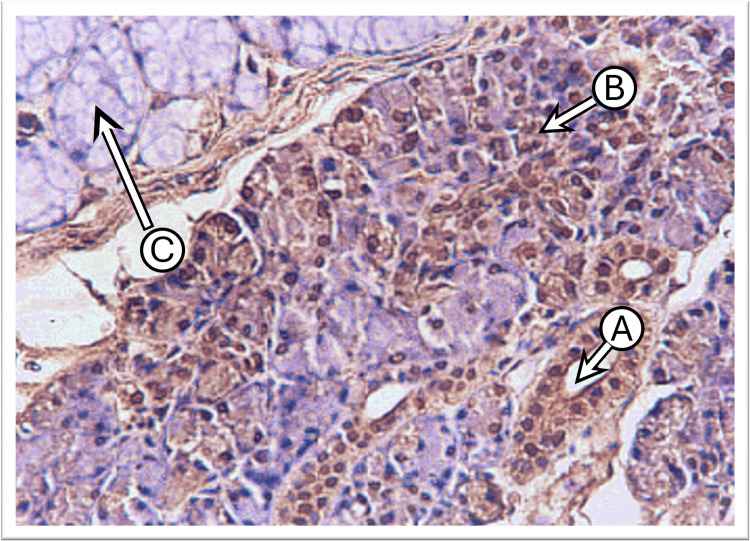
Photomicrograph of the parotid gland subjected to radiotherapy shows strong CK17 staining at the apical part of the duct cells and moderate at their basal part “A,” moderate in serous acini “B,” and negative in mucous acini of the sublingual gland “C” (immunoperoxidase technique x200 magnification)

In the group that received radiotherapy, the parotid gland incubated with anti-cytokeratin E3 antibody against CK17 revealed moderate to strong CK17 expression in the cells of the duct system. Two distinct expression patterns were observed: the prominent pattern was characterized by diffuse expression all over the duct cells, whereas the second showed strong expression at the apical cell part of the duct cells with milder expression at the basal cell part in contrast to the control glands. Additionally, many serous acinar cells displayed moderate to strong diffuse CK17 expression, which was more pronounced than in the control group (Figure 5).

**Figure 6:**
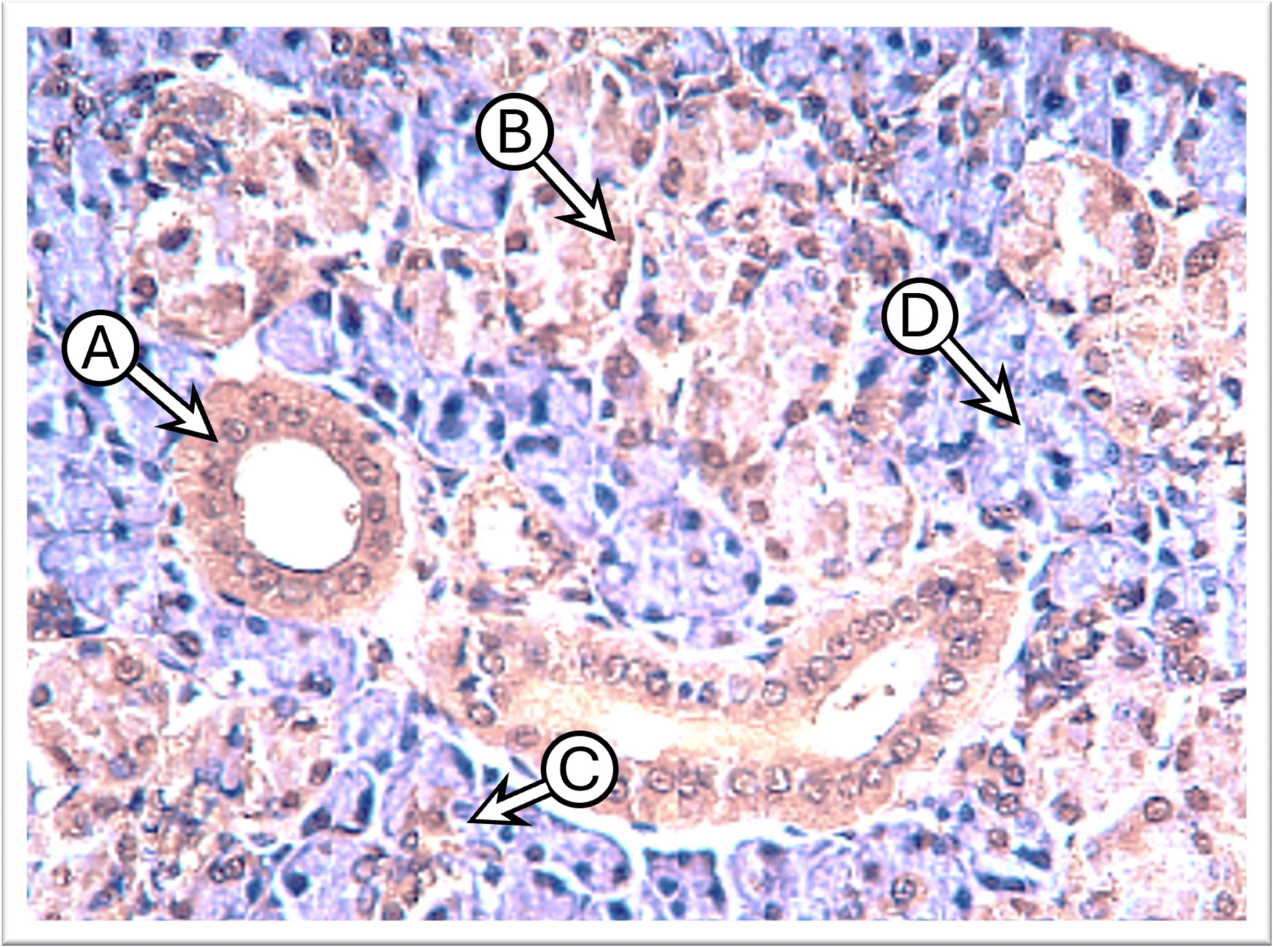
Photomicrograph of the submandibular gland subjected to radiotherapy shows strong CK17 staining at the apical part of the duct cells and moderate at their basal part “A,” mild in granular convoluting tubules “B,” weak in some serous acini “C,” and negative in most serous acini “D” (immunoperoxidase technique x200 magnification)

The examination of the irradiated submandibular gland revealed strong CK17 expression in all intercalated, striated, and excretory duct cells. The staining pattern varied, showing either strong staining in the apical cell part with mild staining at the basal part or diffuse expression throughout the cytoplasm. The main excretory ducts exhibited strong expression in the apical cell layer, with mild expression in the other layers, including the basal layer. Granular convoluted tubular cells lying between interlobular and intralobular ducts displayed diffuse weak to moderate staining. Many serous acini and demilunes exhibited mild to moderate diffuse expression of CK17, while mucous acini showed no staining (Figure 6).

**Figure 7:**
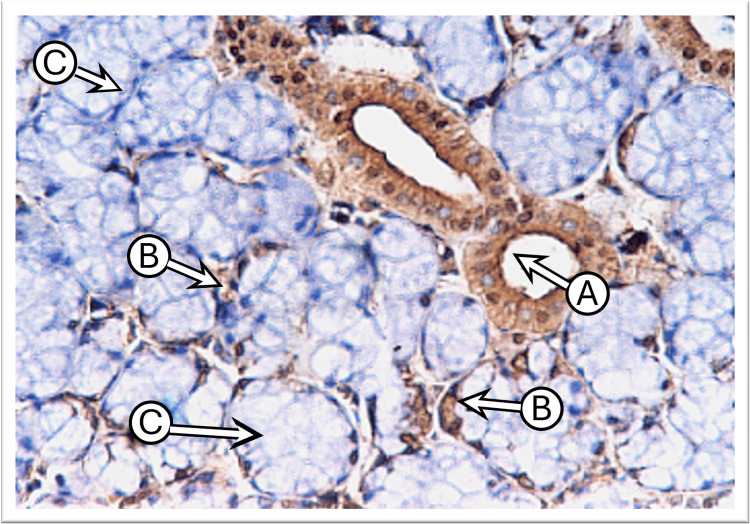
Photomicrograph of the sublingual gland subjected to radiotherapy shows strong CK17 staining at the apical part of the duct cells and moderate at their basal part “A,” moderate in serous demilune “B,” and negative in mucous acini “C” (immunoperoxidase technique x200 magnification)

The examination of the sublingual gland from the irradiated group exhibited moderate to strong diffuse expression of CK17 within the duct system. Occasionally, CK17 expression was more pronounced at the apical region of the cell cytoplasm. The mucous secretory acini did not show CK17 expression, whereas the serous demilunes displayed moderate to strong diffuse staining (Figure 7).

**Figure 8:**
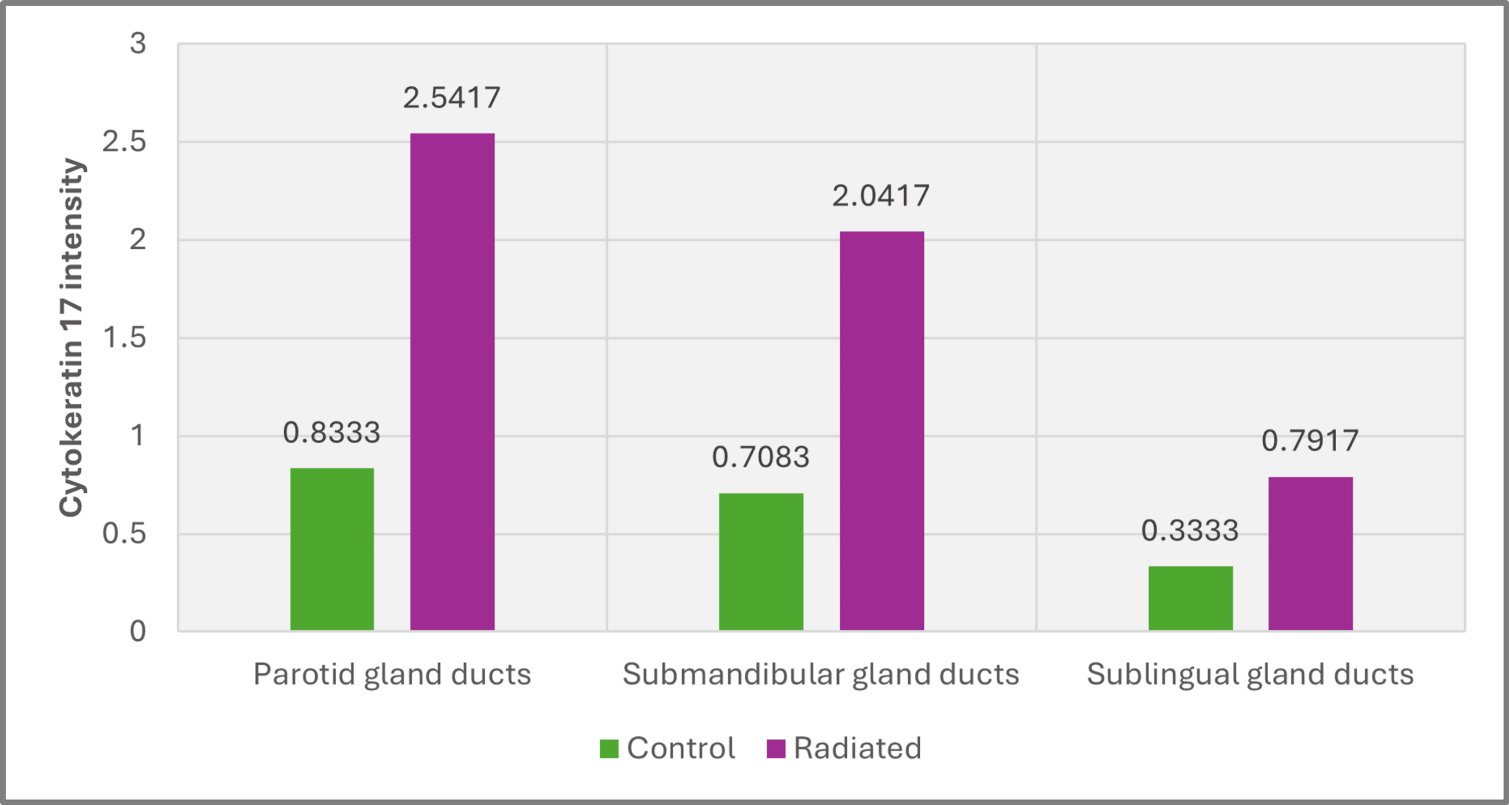
Chart of CK17 intensity in salivary gland ducts of all groups

The statistical analysis indicated that radiation exposure significantly impacted CK17 expression in the duct cells across all major glands, with the differences between the control and irradiated groups being highly significant. Among the glands studied, the submandibular gland exhibited the most pronounced alteration in CK17 expression, with a very low p-value of 0.002, indicating a strong effect of radiation. The parotid gland also showed significant changes (p=0.004), although to a lesser extent than the submandibular gland. In contrast, the sublingual gland demonstrated the smallest impact in CK17 expression with a p-value of 0.032, suggesting that radiation had a relatively minor effect on this gland compared to the others (Figure 8, Tables 1-2).

**Table 1:** Group statistics of CK17 expressions in the duct cells of all groups

	Group	N	Mean	Std. deviation	Std. error mean
Parotid gland ducts	Control	6	0.8333	0.46547	0.19003
	Radiated	6	2.5417	1.02977	0.42040
Submandibular gland ducts	Control	6	0.7083	0.48520	0.19808
	Radiated	6	2.0417	0.64064	0.26154
Sublingual gland ducts	Control	6	0.3333	0.25820	0.10541
	Radiated	6	0.7917	0.36799	0.15023

**Table 2:** Independent samples t-test of CCK17 expressions in the duct cells of all groups

		Levene's test for equality of variances		T-test for equality of means						
		F	Sig.	t	df	Sig. (2-tailed)	Mean difference	Std. error difference	95% confidence interval of the difference	
									Lower	Upper
Parotid gland ducts	Equal variances assumed	3.218	0.103	-3.703	10	0.004	-1.70833	0.46135	-2.73629	-.68037
	Equal variances not assumed			-3.703	6.961	0.008	-1.70833	0.46135	-2.80049	-0.61618
Submandibular gland ducts	Equal variances assumed	0.212	0.655	-4.064	10	0.002	-1.33333	0.32808	-2.06435	-0.60232
	Equal variances not assumed			-4.064	9.316	0.003	-1.33333	0.32808	-2.07169	-0.59498
Sublingual gland ducts	Equal variances assumed	1.038	0.332	-2.497	10	0.032	-0.45833	0.18352	-0.86725	-0.04942
	Equal variances not assumed			-2.497	8.963	0.034	-0.45833	0.18352	-0.87375	-0.04291

**Figure 9:**
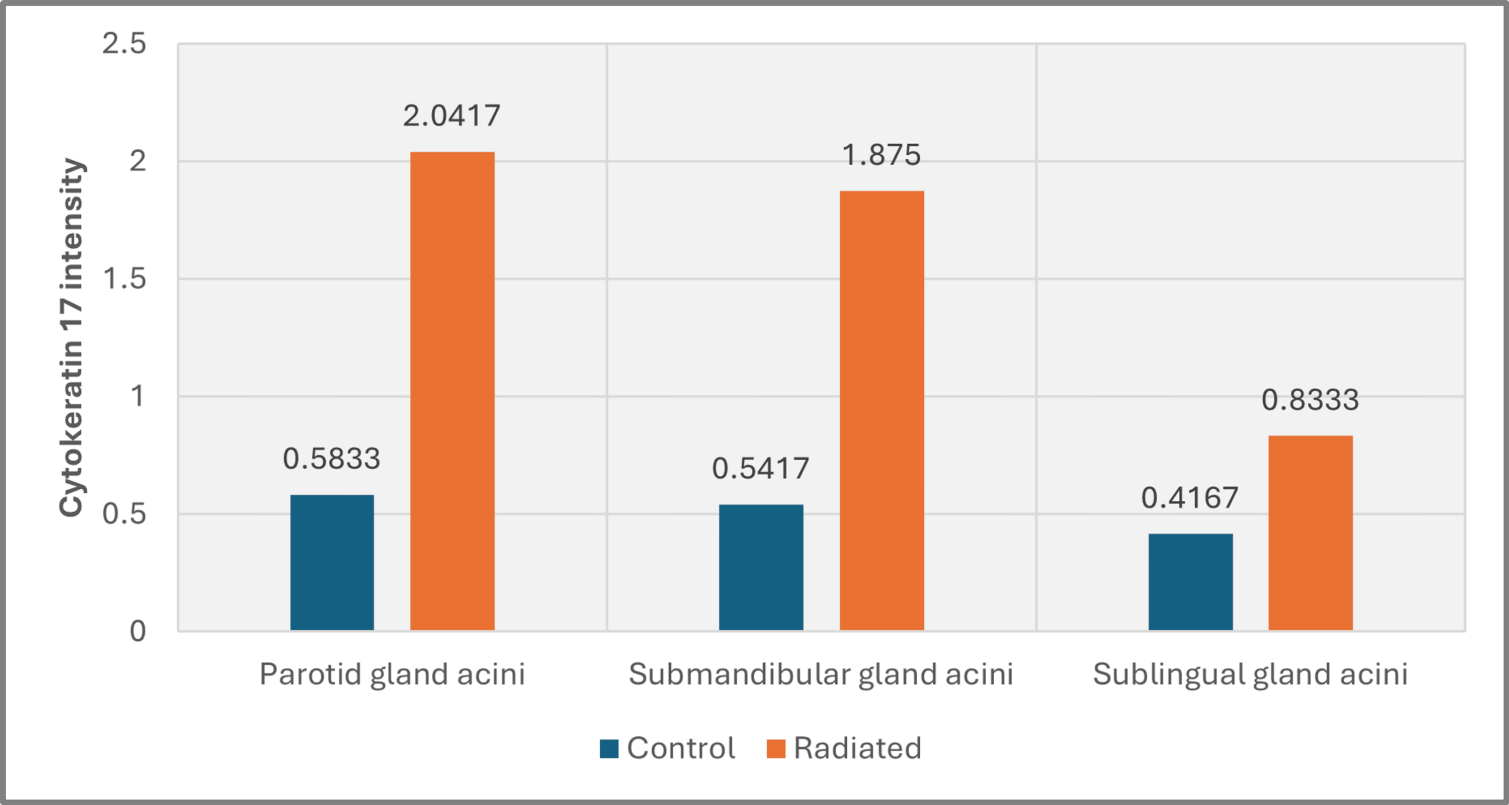
Chart of CK17 intensity in salivary gland acini of all groups

Statistical analysis of CK17 expression in acinar cells revealed significant variation in all glands, with the most pronounced change in the parotid gland (p=0.005), followed closely by the submandibular gland (p=0.006). The sublingual gland exhibited the least effect on acinar cell cytokeratin concentration, although the difference was still statistically significant (p=0.011) (Figure 9, Tables 3-4).

**Table 3:** Group statistics of CK17 expressions in the acinar cells of all groups

	Group	N	Mean	Std. deviation	Std. error mean
Parotid gland acini	Control	6	0.5833	0.46547	0.19003
	Radiated	6	2.0417	0.91401	0.37314
Submandibular gland acini	Control	6	0.5417	0.36799	0.15023
	Radiated	6	1.8750	0.86241	0.35208
Sublingual gland acini	Control	6	0.4167	0.25820	0.10541
	Radiated	6	0.8333	0.20412	0.08333

**Table 4:** Independent samples T-test of CK17 expressions in the acinar cells of all groups

		Levene’s test for equality of variances		T-test for equality of means						
		F	Sig.	t	df	Sig. (2-tailed)	Mean difference	Std. error difference	95% confidence interval of the difference	
									Lower	Upper
Parotid gland acini	Equal variances assumed	2.280	0.162	-3.483	10	0.005	-1.45833	0.41874	-2.39135	-0.52531
	Equal variances not assumed			-3.483	7.430	0.009	-1.45833	0.41874	-2.43701	-0.47966
Submandibular gland acini	Equal variances assumed	3.462	0.092	-3.483	10	0.006	-1.33333	0.38279	-2.18624	-.48043
	Equal variances not assumed			-3.483	6.762	0.011	-1.33333	0.38279	-2.24498	-0.42169
Sublingual gland acini	Equal variances assumed	0.156	0.701	-3.101	10	0.011	-0.41667	0.13437	-0.71606	-0.11727
	Equal variances not assumed			-3.101	9.494	0.012	-0.41667	0.13437	-0.71824	-0.11509

Overall, the findings underscore that radiation exposure has a gland-specific impact, with the submandibular and parotid glands being more vulnerable to changes in CK17 cytokeratin concentration than the sublingual gland. These results provide insight into the differential response of salivary glands to radiation, potentially guiding future research on long-term glandular radiation damage and its underlying mechanisms.

## Discussion

Xerostomia is the most common and debilitating side effect of radiotherapy for HNMs resulting from damage to the salivary gland tissue. While this effect is well-documented in humans and experimental animals, the mechanisms underlying radiation-induced changes remain poorly understood.

The current study found that radiotherapy caused structural changes, including atrophy of the parenchymal elements with the proliferation of fibrous and fatty tissue, which impair the gland's function. These findings are consistent with those of several other studies [15,18,22]. Paardekooper et al. (1998) identified early radiation-induced alterations in parenchymal tissue as a key factor contributing to the acute loss of gland function. They found that this effect is dose-dependent, with even low doses of radiation being capable of causing cellular damage and dysfunction [22]. In contrast to the atrophic changes observed in the parenchymal elements in our study, the fibrous stroma showed signs of proliferative activity, emphasizing the different impacts of irradiation on connective tissue compared to epithelial tissue. The dense fibrous tissue that replaced the degenerated parenchymal components suggests permanent structural changes, which may hinder the glands' ability to regenerate.

The presence of both normal and reduced acini in the major irradiated glands of our study indicates that the gland continues to perform its secretory activity, though at a diminished capacity. Numerous studies have reported that radiation therapy-induced damage to the salivary glands leads to irreversible dysfunction in 63-93% of patients, particularly when the cumulative dose exceeds 30 Gy. The permanent nature of this damage is concerning, as it diminishes the likelihood of recovery or restoration of normal gland function after radiation therapy [23-25]. However, it is important to note that while a significant percentage of patients experience permanent damage, there remains a chance for complete salivary gland recovery. Additionally, Mata et al. (2004) proposed that the persistent acini in the irradiated gland tissues could contribute to the gland's regenerative capacity [26]. In the current study, several specimens from irradiated glands displayed large cells with lightly vacuolated cytoplasm and enlarged nuclei, along with the continued presence of abnormal mitosis. The presence of abnormal mitosis in some of these cells suggests the potential development of neoplastic tissue, consistent with findings reported by various researchers [27-29]. This observed feature appears to result from the damaging effect of radiation on DNA replication in parenchymal cells, ultimately leading to cell death. This finding is consistent with the conclusions of many researchers [30-31]. Krishnan et al. (2017) observed that cytoplasmic vacuolation is associated with the impact of irradiation on the cell membrane, leading to cellular irregularities and a reduction in amylase secretion [32].

Cytokeratin is a type of intermediate filament protein found in epithelial cells that plays a crucial role in the structural integrity of both cells and tissues, and its expression is closely associated with the differentiation status [33]. Immunohistochemical profiling revealed that all control major glands exhibited mild to moderate CK17 in the duct cells with weak expression in the serous cells. The increased expression of CK17 in the duct system relative to the serous acini may suggest that more differentiated acinar cells have fewer filamentous structures, potentially aiding in the facilitation of saliva secretion. Multiple studies support the finding that CK17 in salivary gland cells is crucial for cell structure, with the level of expression closely associated with the differentiation status of the parenchymal cells [5,15,34]. The different distribution patterns of cytokeratin observed in our results are thought to correlate with the active gland function. A diffuse pattern refers to the resting state, while the decreased cytokeratin expression in the apical portion is associated with exocytosis, which agrees with many authors [35]. The concentration of cytokeratin at the basal cell part may contribute to increased tensile strength in the region facing the myoepithelium. This increased strength could enhance the squeezing action, facilitating the movement of saliva through the lumen.

The immunohistochemical analysis of the group that underwent radiotherapy showed a notably stronger staining reaction in the duct system and serous acini; these results agree with many authors [15,34,35]. One possible explanation is that radiation rapidly disrupts intermediate filaments, causing their components to aggregate into filament bundles that accumulate in the cytoplasm. This accumulation obstructs saliva production by interfering with the endoplasmic reticulum and Golgi apparatus, hindering the saliva transport pathway and preventing its progression to exocytosis. In most sections of the irradiated group, strong cytokeratin expression was observed in the luminal region of the ductal cell cytoplasm, decreasing towards the basal area. This distribution pattern could disrupt ductal function, impairing their ability to modify salivary secretion. Additionally, diffuse cytokeratin expression throughout the cell may indicate widespread cellular damage. The luminal pattern is thought to represent an early stage of radiation-induced damage, while the diffuse pattern suggests a more advanced stage of injury. Lastly, the lack of cytokeratin staining in the mucous acini of both groups suggests that CK17 may not play a role in their structural or functional properties, potentially indicating a different intermediate filament profile in mucous acinar cells.

Although the results of the current study provide valuable insights, I believe that increasing the sample size would have strengthened the statistical analyses and yielded more accurate results. Additionally, a time sequence from the onset of radiation lasting at least three months is necessary to fully understand the pathological effect curve and determine when the effects begin to stabilize.

## Conclusions

The positive immunostaining reaction of the control salivary glands suggests that small amounts of intermediate filaments are necessary for saliva secretion. Radiotherapy to the head and neck significantly affects the major salivary glands, disrupting cytokeratin arrangement and density, which impairs saliva production. The most noticeable changes were observed in the submandibular gland, followed by the parotid gland, with the sublingual gland showing the least impact. However, CK17 intensity in acinar cells was highest in the parotid gland, then the submandibular gland, with the least in the sublingual gland. Further studies are needed to explore the short- and long-term effects to determine if these glands can fully recover over time.
